# The influence of the quantity and quality of sediment organic matter on the potential mobility and toxicity of trace elements in bottom sediment

**DOI:** 10.1007/s10653-019-00359-7

**Published:** 2019-06-24

**Authors:** Agnieszka Baran, Monika Mierzwa-Hersztek, Krzysztof Gondek, Marek Tarnawski, Magdalena Szara, Olga Gorczyca, Tomasz Koniarz

**Affiliations:** 1 Department of Agricultural and Environmental Chemistry, University of Agriculture in Krakow, Al. Mickiewicza 21, 31-120 Kraków, Poland; 2 Department of Hydraulic Engineering and Geotechnics, University of Agriculture in Krakow, Al. Mickiewicza 24/28, 30-059 Kraków, Poland

**Keywords:** Bottom sediments, Trace elements, Fraction of organic matter, Mobility, Toxicity

## Abstract

Knowledge on the fraction of trace elements in the bottom sediments is a key to understand their mobility and ecotoxicological impact. The purpose of this study was to assess the influence of the content of organic matter fractions on the mobility and ecotoxicity of trace elements in sediments from the Rybnik reservoir. The most refractory fraction of organic matter—Cnh (non-hydrolysing carbon)—dominated in the sediments. The content of organic matter fractions are arranged in the following order: Cnh (non-hydrolysing carbon) > Cfa (fulvic acid) > Cha (humic acid) > DOC (dissolved organic carbon). On the other hand, the highest value of correlation coefficients was found for different fractions of trace elements and DOC content in the bottom sediments. A higher content of TOC in the sediments significantly increased the share of elements in the potential mobile fraction and, at the same time, decreased the binding of elements in the mobile fractions. Moreover, in sediments that contain more than 100 g/kg d.m. TOC, no and medium risk of trace element release from sediments was observed. The Cu, Cd and Ni were potentially the most toxic elements for biota in the Rybnik reservoir. However, the correlation between the content of trace elements and the response of bacteria was insignificant. These results suggested that the complexation of trace elements with organic matter makes them less toxic for *Vibrio fischeri*. The transformation and sources of organic matter play an important role in the behaviour of trace elements in the bottom sediments of the Rybnik reservoir.

## Introduction

Among the contaminants of bottom sediments, a significant role is played by trace elements, which, given certain contents and conditions, are characterised by toxicity towards living organisms, bond durability as well as the ability of activation at different stages of the food chain (Rosado et al. 2016a, b; Tarnawski and Baran 2018). Trace elements that have accumulated in bottom sediments can potentially be toxic to aquatic organisms. Moreover, in the case of improper sediment management, e.g. from the dredging of reservoirs, they can also pose a threat of having a toxic impact on land organisms. In order to determine the ability of contaminants to migrate within the environment, particularly considering the inclusion of contaminants in biogeochemical cycles, it is necessary to assess the reactivity and mobility of those compounds (Farkas et al. 2007; Rosado et al. 2016a, b; Gao et al. 2018). In order to assess the environmental threat related to trace elements accumulated in bottom sediments, it is adequate to determine the share of their forms that comprise the overall content (Martínez-Santos et al. 2015). It is especially useful to possess knowledge on the content of the forms of elements that are easily dissolvable or exchangeable, considering their ability to activate from the solid state and migrate to the aquatic environment, where they become biologically available (Sutherland and Tack 2007; Baran and Tarnawski 2015). Sequential chemical extraction is considered as an important source of information on the chemical bonds in which trace elements occur in bottom sediments. This is also reflected in the mechanisms of their behaviour in the environment, among others, in mobility, toxicity and potential bioavailability (Rinklebe and Shaheen 2014; Baran and Tarnawski 2015). The behaviour of trace elements in bottom sediments is controlled by many factors, such as: pH, redox condition, temperature, electric conductivity, content of the clay fraction, content of iron and manganese oxides and the form of elements (Singh et al. 2005; Fonseca et al. 2013; Cao et al. 2015; Martínez-Santos et al. 2015). However, the content and quality of organic matter play a very important role in the assessment of trace element behaviour in the aquatic environment (Filcheva and Yurukova 2004; Hristov et al. 2009; Yang et al. 2011; Smith et al. 2014; Smal et al. 2015; Derrien et al. 2017). The organic matter of bottom sediments is subjected to various processes, which may affect its character in a relatively short period of time, leading to the creation of humic compounds that are active in reactions with trace elements (Derrien et al. 2017). Moreover, as a result of decomposition, organic matter may constitute a source biogenic compound, and as a result of transformations, it may have a significant effect on the mobility, bioavailability and toxicity of trace elements (Filcheva et al. 2014; Bai et al. 2018). Trace elements can be bound by organic matter as a result of exchange sorption, complexation or chelation. The effectiveness of organic matter in limiting the mobility of trace elements is conditioned by its properties and degree of transformation.

The aims of the study were: (1) to investigate the content of organic matter fractions in bottom sediment, (2) to assess the mobility and potential bioavailability of trace elements in sediments by using chemical fractionation, (3) to determine the contribution of organic matter in the binding of trace elements, and (4) to assess the ecotoxicity of bottom sediments using the *Vibrio fischeri* biotest. The evaluation of the interaction between fractions of organic matter and trace elements as well as the response of organisms is useful for a justified pollution risk assessment.

## Materials and methods

### Study area and sediment sampling

**Fig. 1:**
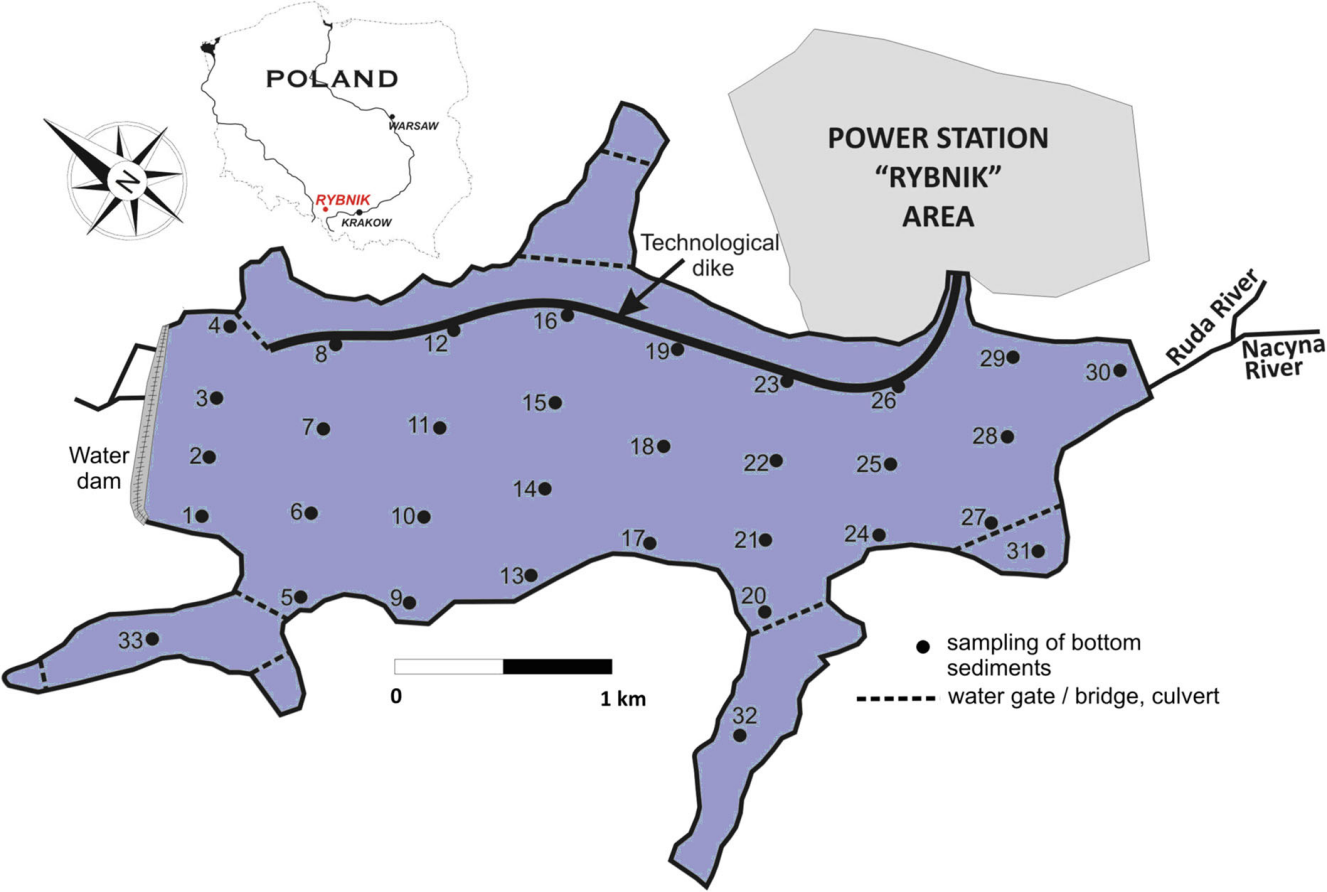
Location of the Rybnik reservoir and bottom sediment sampling points

The Rybnik reservoir is located in the Silesian Voivodeship, in the centre of the Rybnik Coal Area (50°8′26N, 18°29′51E), which is one of the main industrial centres of southern Poland. Silesia is a highly urbanised region, which also concentrates industry (mainly hard coal mining), power generation, metallurgy and transport, as well as the production of machinery, chemicals and building materials. The Rybnik reservoir is a dam reservoir constructed on the Ruda river (south-western Poland) in the 1970s (Fig. 1). The Ruda river is a tributary of the Oder river. It is 50.6 km long and its basin covers an area of 416.4 km^2^, generating an average flow of approx. 3.3 m^3^ s^−1^. It is worth adding that the Ruda river, as well as its tributary—Nacyna—are one of the most contaminated rivers in Poland. The basic morphometric data of the Rybnik reservoir are as follows: area of the main reservoir—4.44 km^2^, length—4.5 km, maximum capacity—24 million m^3^, normal capacity—22.5 million m^3^, average depth—approx. 5 m. The reservoir has four side bays with limited mixing of water masses as a result of existing culverts and bridges along various transport routes. The Rybnik reservoir serves several important functions: technological, storage and recreational. It is important that the reservoir constitutes a part of the technological chain of the Rybnik power plant as an essential source of cooling water as well as direct receiving water of treated industrial sewage (Baran and Tarnawski 2015; Kostecki et al. 2017).

The samples were collected from 33 stations (Fig. 1), from under the surface of the water. The sample extraction points were previously planned in such a location that the main part of the reservoir would be evenly covered. The lateral bays are represented by individual samples due to the influence of external sources and the limitation (bridges) of contact with potential major sources of pollution (Ruda river, power plant) in the main part of the reservoir. The top layer of the sediment was collected from a depth of 0–15 cm using an Ekman sampler. In justified cases, underwater sampling was carried out by divers when the sediments contained many woody debris that prevented the sampler jaws from closing. After the decantation of overlying water, all sediment samples were refrigerated until analysed.

### Chemical analysis

#### Basic physicochemical properties of bottom sediments and organic matter fractions

The properties of the bottom sediment, which have been analysed, are as follows: particle size fractions, pH and redox potential. The analytical methods, as well as the above parameters, have been described in our previous studies (Tarnawski and Baran 2018). The content of total organic carbon (TOC) in sediments was determined using a CNS analyser (Vario EL Cube, Elementar Analysensysteme 2013). The content of humus compounds was extracted from bottom sediments using a mixture of 0.1 mol dm^−3^ Na_4_P_2_O_7_ solution and 0.1 mol dm^−3^ NaOH (Mierzwa-Hersztek et al. 2018). The carbon of humic acids (Cha) was isolated in the extract of sodium pyrophosphate and a sodium base, whereas the carbon of fulvic acids (Ckf) was calculated from the difference between the amount of carbon (C ext) as well as the amount of humic acid carbon (Cha) in the extract. The extraction residue—non-hydrolysing carbon (Cnh)—was computed from the difference between the total organic carbon content (TOC) and the amount of carbon in the extract. In the prepared solutions of humic acids, light absorbance was measured at the 465 and 665 nm wavelength and the colour ratio (*E*_4_/*E*_6_) was computed (Mierzwa-Hersztek et al. 2018). In order to determine the dissolved organic carbon (DOC), the sediment samples were extracted in sediment: water ratio 1:10 v/v, by shaking on a rotary shaker for 24 h. Next, the samples were centrifuged in 50-ml tubes at 3000×*g* for 10 min, and filtered through a 0.45 μm membrane filter (Akkanen et al. 2005). The DOC content was measured using TOC analyser 1200 (Thermo Elektron).

#### Fraction of trace elements

An analysis of the fractionation of trace elements (Zn, Cd, Pb, Cu, Ni, Cr, As) in the bottom sediments was conducted with the use of sequential chemical extraction modified by the BCR method (BCR 2001; Sutherland and Tack 2007; Martínez-Santos et al. 2015). The following four fractions of trace elements have been distinguished: exchangeable (fraction 1—ion exchange and carbonate), related to hydrated Fe oxides as well as Mn oxides (fraction 2—reduction), related to organic matter (fraction 3—oxidation), as well as elements permanently bound with minerals (fraction 4—residual) (Baran and Tarnawski 2015). After each step, the extracts were separated from the solid residue by centrifugation at 4500 rpm for 5 min, and the supernatant liquids were decanted into a polyethylene container and analysed for the concentration of trace elements. The residues were washed by adding 30 ml of distilled water, shaken for 15 min, centrifuged and then the obtained extract was discarded. The residual content of trace elements in the sediments was denoted by dissolving the material using the “wet” method in a closed system using the AntonPaar Multiwave 3000 microwave system in a mixture of HNO_3_/HCl acids (3:1 v/v) (suprapure, MERCK). The determination of trace elements in the acquired extracts was conducted using an atomic emission spectrophotometer—Optima 7300 DV supplied by PerkinElmer—with inductively coupled plasma (ICP-OES). The assessment of the analytical performance was conducted using reference material BCR-701. The results showed that the percentage of recovery ranged from 80 to 119% for Zn, from 79 to 122% for Cu, from 91 to 124% for Pb, from 79 to 118% for Cr, from 85 to 112% for Cd and from 85 to 128% for Ni.

### Ecotoxicity assessment

The ecotoxicity of the bottom sediment was assessed using a bacterial biotest—Microtox. The inhibition of luminescence *Vibrio fischeri* was the endpoint in this test. In order to test the sediment samples, 81.9% Screening Test and the Microtox M500 Analyser (Microbics Corporation 1992) were applied. The test method was described in our earlier studies (Baran and Tarnawski 2015). The toxicity assessment developed by Persoone et al. (2003) was used to evaluate the sediment toxicity: PE (Per cent toxic effect) < 20% no toxic effect; 20% ≤ PE < 50% low toxic sample; 50% ≤ PE < 100% toxic sample, PE-100% very toxic sample.

### Statistical and graphical analysis

The results were verified statistically using the STATISTICA 12 software package. The statistic variables included the mean, standard deviation, minimum, maximum and the coefficient of variation (CV %). The differences between the means were analysed by ANOVA at a significance level of 0.05. Pearson’s correlation, principal component analysis (PCA) and cluster analysis were preformed to establish the relationship and behaviour of trace elements and organic matter fractions in the bottom sediments. Variability maps were created using Surfer 8.0 software.

## Results

### Basic characteristic of bottom sediments and organic matter fractions

**Fig. 2:**
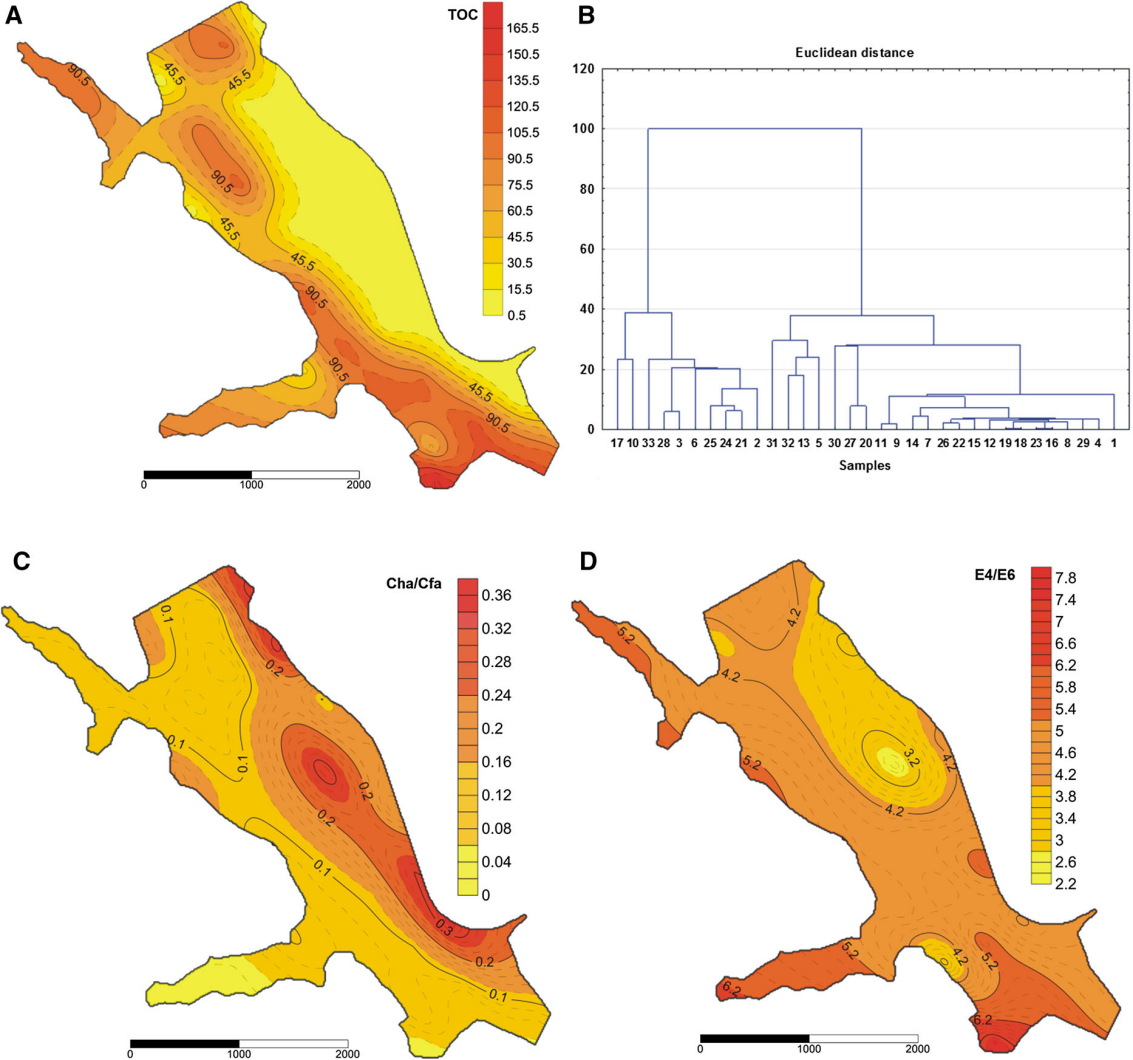
Spatial distribution of TOC (g/kg d.m.), Cha/Cfa, *E*_4_/*E*_6_ and cluster analysis of fraction of organic matter according to their spatial distribution in the bottom sediments. **a** TOC, **b** cluster analysis, **c** Cha/Cfa, **d***E*_4_/*E*_6_

Based on the particle size fraction, the sand fraction was dominant in the bottom sediments sampled from the Rybnik reservoir. The bottom sediments contain 48 to 99% sand and 1 to 52% mud (silt + clay). The bottom sediments revealed a weak acid, neutral and alkaline reaction, and the pH ranged from 6.59 to 8.53. The redox potential (Eh) ranged from 103 to 11.4. The denoted TOC values in the studied bottom sediments differed significantly (2.04–166.05 g/kg s.m., CV = 102%) within the reservoir, which was the result of the location of sample collection and likely the depth of the reservoir in places where the bottom sediment was collected (Table 1, Fig. 2). Longitudinal zonation in the TOC spatial distribution was demonstrated in the Rybnik reservoir. The lowest TOC content was found in the sediments collected in the right-bank part of the Rybnik reservoir, while the highest content was found in the left-bank part of the reservoir (Fig. 2). Such a significant diversification in the content of TOC was not confirmed by the results of research conducted by Cieślewicz et al. (2008) and Smal et al. (2015). On the other hand, Szymański et al. (2016), Hou et al. (2014) and Mazzuoli et al. (2003) state that the considerable diversification in the content of TOC in the studied bottom sediment is not only the result of the location of the reservoir from which the research material was collected, but that it can also be dictated by the date on which the research material was collected, as well as intensive anthropogenic activity. The results found three groups of sediment samples significant, which differed in the TOC content (significant differences at *p* ≤ 0.05). A high content of TOC (> 100 g/kg d.m.) was exhibited by sediment samples collected at points: 31, 28, 17, 24, 10, 21, 30, 3, 25, 6, 33; medium content (10–100 g TOC/kg d.m.): 2, 32, 27, 13, 5, 20, 9, 14, 7 as well as low content (< 10 g TOC/kg d.m.): 12, 11, 22, 1, 19, 18, 8, 16, 29, 4, 23, 26, 15 (Figs. 1, 2). It should also be emphasised that the denoted TOC contents in the studied bottom sediments were significantly lower than the contents that can be registered for bottom sediments from lakes (Punning and Tougu 2000), as well as significantly higher than those observed for bottom sediments in the case of dam reservoirs. The bottom sediments of the Chańcza reservoir contained on average 35.4 g, the Besko reservoir—18.2 g, the Zesławice reservoir—15.8 g, the Rzeszów reservoir—23.5 g, the Ożanna reservoir—31.3 g TOC/kg d.m. (Baran and Tarnawski 2013; Baran et al. 2017). The content of extracted carbon (Cext) in a mixture of Na_4_P_2_O_7_ + NaOH with a concentration of 0.1 mol dm^−3^ exhibited less diversification in comparison with the TOC content (*V*% = 86) (Table 1). The average share of Cext in the TOC content was almost 24%. The content of carbon in the humic acid fraction (Cha) was over ten times smaller than the content of carbon in the fulvic acid fraction (Cfa) (Table 1). Hou et al. (2014) and Khodorenko et al. (2012) also observed a 1.5–3 times higher content of Cfa than of Cha in the bottom sediment. The predominance of the Cfa fraction over the Cha fraction can be associated with alkaline environments in the reservoir, as the mean value of the water pH in the Rybnik reservoir was 8.05. These conditions favour the formation of fulvic acids. The average share of the Cha content in the TOC content was the highest for bottom sediment samples characterised by a TOC content of not more than 10 g/kg d.m. It has to be noted that both the content of Cha and Cfa in the content of TOC decreased along with an increase in the TOC content. We suggest that the above relationships are associated with an intensive accumulation of poorly decomposing macrophyte tissues in the bottom sediments, which decreased the contribution of humus substances in the TOC content. The contents of carbon accumulated in the Cha and Cfa fractions were reflected in the value of the Cha/Cfa ratio, which did not exceed unity and was shaped from a relatively low value of 0.02–0.35 (Fig. 2c). As can be seen from the spatial layout of the Cha/Cfa values, larger values of this factor were characterised for the right-side bank of the reservoir, which can be dictated by the erosion of the shoreline. The content of carbon that accumulated in the Cnh fraction was on average 39.26 g/kg d.m. and exhibited the highest diversification among the analysed fractions of organic matter of bottom sediments (Table 1). It was demonstrated that the share of Cnh in the content of TOC increased along with an increase in the TOC content, reaching a peak share (almost 80%) in samples characterised by a content exceeding 100 g TOC/kg d.m. In the studies of Fonseca et al. (2013), Cnh contents (32.9–82.6 g/kg d.m.) were also predominated in the fractions of organic matter. Changes in the optical density of humic acid solutions extracted from bottom sediment samples were expressed by values of the absorption ratio: *E*_4_/*E*_6_. In general, in the analysed material, values of the *E*_4_/*E*_6_ ratio lower than 5 were noted (Fig. 2d). Studies conducted by Derrien et al. (2017) as well as Tadini et al. (2015) show that the development of catchment has a significant effect on the properties of humic acids. These differences do not only concern the elemental composition, but they may also concern spectral and thermal properties. According to the cited authors, humic acids created in the lakes of forest catchments are characterised by higher values of the *E*_4_/*E*_6_ ratio, which means that they contain a higher number of aromatic structures than humic acids isolated from the bottom sediments of lakes in agricultural catchments. The acquired values of the *E*_4_/*E*_6_ ratio for the extracted humic acids were comparable to the values obtained by Giovanela et al. (2010). An analysis of the spatial layout of the values of the *E*_4_/*E*_6_ ratio shows that humic acids extracted from bottom sediments collected on the right-side bank of the reservoir exhibit more aromatic structures. It was proven that particles of humic substances derived from land are characterised by a higher degree of aromaticity due to a large share of highly aromatic fragments from lignins (Tadini et al. 2015). On the other hand, humic substances of aquatic origin have a larger share of aliphatic carbon and a lower share of aromatic groups (Cieslewicz et al. 2008). In the studies of Mengchang et al. (2008), the highest aromaticity of humic acid in the bottom sediments from a river located in the moderate and cold zone and the lowest aromaticity in the sediments of a eutrophic lake were observed. The content of dissolved organic carbon (DOC) is an important indicator when characterising bottom sediments due to its high significance in the assessment of water quality. DOC is considered to be a mobile, available and ecologically important fraction of organic matter in the bottom sediments (Akkanen et al. 2005; Smith et al. 2014). The content of DOC in the studied bottom sediments was on average 0.44 g/kg s.m., which was less than 1% of the TOC content (Table 1). It should also be emphasised that there was significant diversification (*V*% = 100) of the content of this component in the studied bottom sediments. The highest dissolved organic carbon content was found in samples 2, 6, 3, 28, 10, 17, indicating an intense inflow of organic matter in this part of the reservoir (Figs. 1, 2a).

**Table 1:** Content of carbon in the fractions of organic matter in the bottom sediments

Parameter	Mean ± SD	Minimum	Maximum	CV
	g/kg d.m.			%
TOC	51.6 ± 52.5	2.04	166.1	102
C extracted (Cext)	12.3 ± 10.6	1.72	35.2	86
C humic acid (Cha)	0.87 ± 0.53	0.16	2.28	66
C fulvic acid (Cfa)	11.5 ± 10.1	1.32	33.0	88
C non-hydrolysing (Cnh)	39.2 ± 42.7	0.08	143.2	109
DOC	0.44 ± 0.43	0.07	1.29	100

A hierarchical cluster analysis was performed for the grouping of sampling points, which have similarities of distribution in fractions of organic matter (Fig. 2b). The analysis showed a dendrogram where all 33 sites have been grouped into three clusters (Fig. 2b). Cluster 1 includes sampling points 17, 10, 33, 28, 3, 6, 25, 24, 21, 2 and can be generally assigned with a high content of TOC (expect point 2). Cluster 2 includes sites 31, 32, 13, 5, 30, 27, 20 and cluster 3 contains points 1, 4, 29, 8, 16, 23, 18, 19, 12, 15, 22, 26, 7, 14, 11, 9. The sampling points falling into cluster 2 (expect points 31, 30) and cluster 3 (expect points 9, 7, 14) can be considered as having a moderate and low content of TOC, respectively. The dendrogram confirmed that the sampling points of the western part of the reservoir are richer in TOC than the samples on the eastern part (Fig. 1). Moreover, the content and spatial distribution of DOC in the bottom sediment played an important role in the grouping of sampling sites into clusters.

### Trace element fractionation in the bottom sediments

**Fig. 3:**
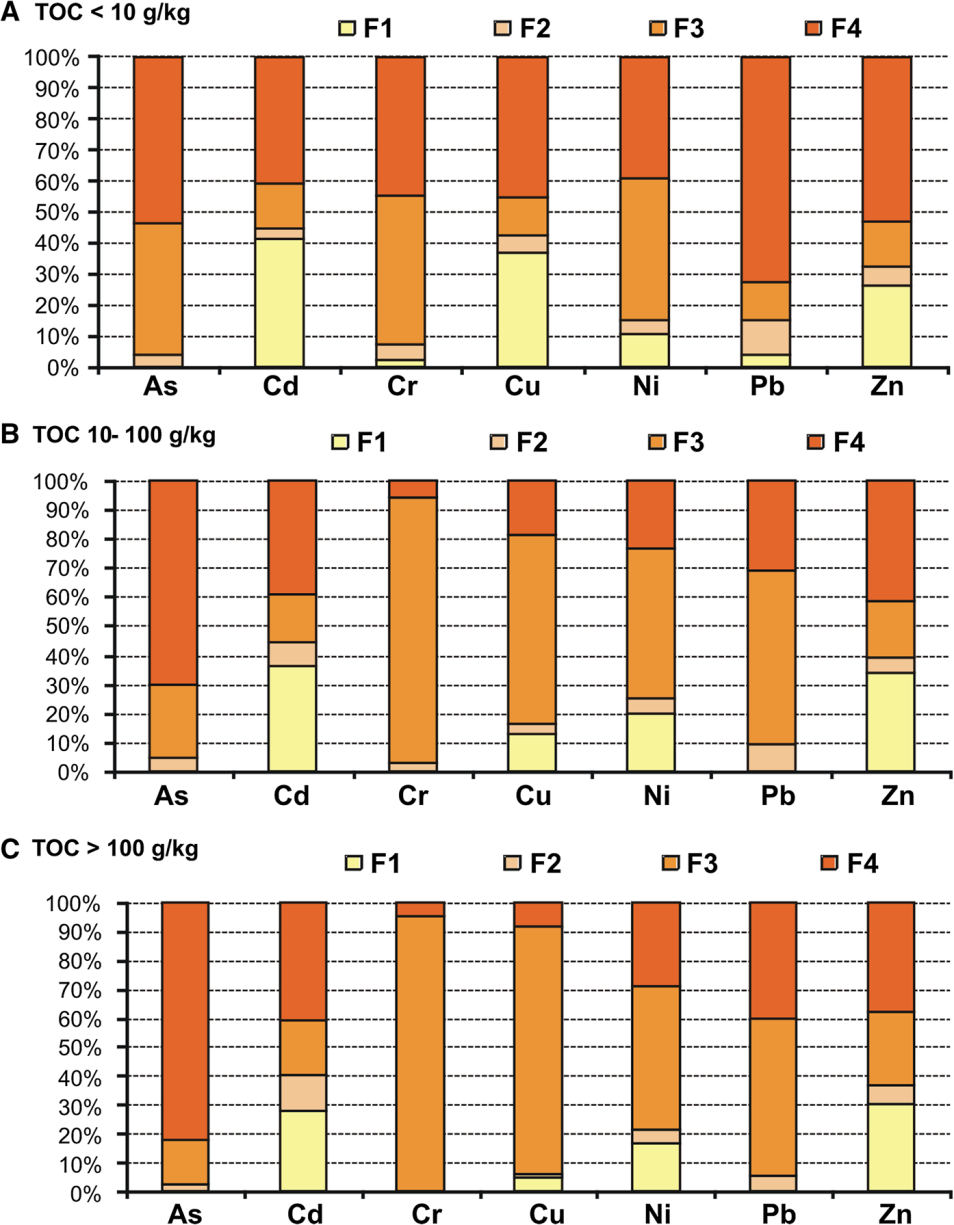
Effect of TOC content on fractional distribution of trace elements in the sediments. **a** TOC < 10 g/kg, **b** TOC 10–100 g/kg, **c** TOC > 100 g/kg

Information on the distribution of trace elements among different fractions in bottom sediments and their behaviour under changing TOC content in sediments was shown in Table 2. The results of the sequential extraction identified different groups of trace elements according to their distribution in fractions depending on the TOC content in the sediments (Fig. 3a–c). Moreover, depending on the content of TOC, we indicated significant differences (*p* ≤ 0.05) between the distributions of trace elements in their chemical fractions (Fig. 3a–c). One of the groups is composed of Cu, Cd and Zn. The greatest proportion of those metals in the samples with a low content of TOC (< 10 g/kg) was mainly associated with F1 (Cd 42%, Cu 37% and Zn 26%) (Fig. 3a). This fraction is bioavailable and can be toxic to aquatic organisms. The second group includes Cr, Cu, Ni and Pb, which are found predominately in F3 (organic), especially in sediment samples with a medium and high TOC content (Table 2, Fig. 3b, c). Depending on content of TOC in the sediments, those elements associated in F3 constituted 48–94% (Cr); 12–85% (Cu); 13–55% (Pb); and 46–50% (Ni), indicating that organic carbon and sulphides are the main agent for those elements, followed by a residual fraction 6–45% (Cr); 8–45% (Cu); 40–72% (Pb); and 23–40 (Ni) their total content in the sediments. In the study of Martínez-Santos et al. (2015), these metals were predominantly bound in fraction 3 (38.4–71.1%). The third group was composed of As (Table 2, Fig. 3a–c). The dominant content of this element was connected with the residue fraction (F4), and depending on the TOC content, it was between 55 and 82%. However, for Cd and Zn, fraction 4 also played an important role in the binding of these metals (Cd 39–41%; Zn 38–53%). In the studies, a generally low content of trace elements (Cd, Cu, Ni, Zn) was found in F2 (Table 2), which suggests that Mn and Fe oxides/hydroxides do not play a dominant role in the sorption of these elements.

**Table 2:** Assessment of mobility of trace elements from sediments

Fraction *n* = 33	Content of trace elements in different fractions (mg/kg d.m.)						
	As	Cd	Cr	Cu	Ni	Pb	Zn
F1	0.03 ± 0.06	1.45 ± 1.21	0.22 ± 0.08	56.13 ± 36.4	4.02 ± 3.54	0.45 ± 0.25	177.5 ± 158
F2	0.20 ± 0.25	0.51 ± 0.61	0.48 ± 0.24	14.35 ± 13.1	1.20 ± 0.97	2.55 ± 2.72	35.75 ± 33.5
F3	1.62 ± 1.03	0.84 ± 0.96	30.06 ± 33.9	462.6 ± 681	12.18 ± 8.97	21.57 ± 23.1	132.30 ± 134
F4	6.08 ± 6.56	1.89 ± 1.80	2.91 ± 4.15	84.37 ± 107	7.21 ± 6.58	16.88 ± 13.5	235.91 ± 187

TOC (g/kg)	RAC classification						
	As	Cd	Cr	Cu	Ni	Pb	Zn
< 10	No^a^	High	Low	High	Medium	Low	Medium
10–100	No	High	No	Medium	Medium	Low	High
> 100	No	Medium	No	Low	Medium	No	Medium

TOC (g/kg)	ICF						
	As	Cd	Cr	Cu	Ni	Pb	Zn
< 10	0.96 ± 1.92	1.92 ± 1.31	1.57 ± 1.08	1.37 ± 0.64	2.57 ± 2.49	0.41 ± 0.13	1.22 ± 0.54
10–100	0.35 ± 0.13	1.50 ± 0.34	15.7 ± 12.6	7.54 ± 9.85	4.99 ± 5.05	1.54 ± 0.88	1.40 ± 0.39
> 100	0.20 ± 0.13	1.50 ± 0.33	21.8 ± 14.5	38.0 ± 43.5	2.95 ± 1.73	1.61 ± 0.87	1.69 ± 0.44
Mean	0.58 ± 1.32	1.69 ± 0.92	11.3 ± 13.5	14.9 ± 31.7	3.21 ± 3.06	1.05 ± 0.86	1.41 ± 0.51

^a^Elements in MF [%]: < 1 no risk; 1–10 low risk; 11–30 medium risk; 31–50 high risk; 50 > very high risk (Singh et al. 2005)

**Fig. 4:**
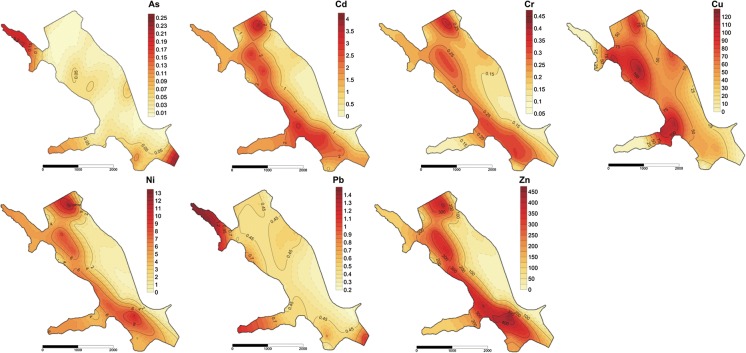
Spatial distribution of mobile fraction (F1) of trace elements (mg/kg d.m.) in bottom sediments

**Fig. 5:**
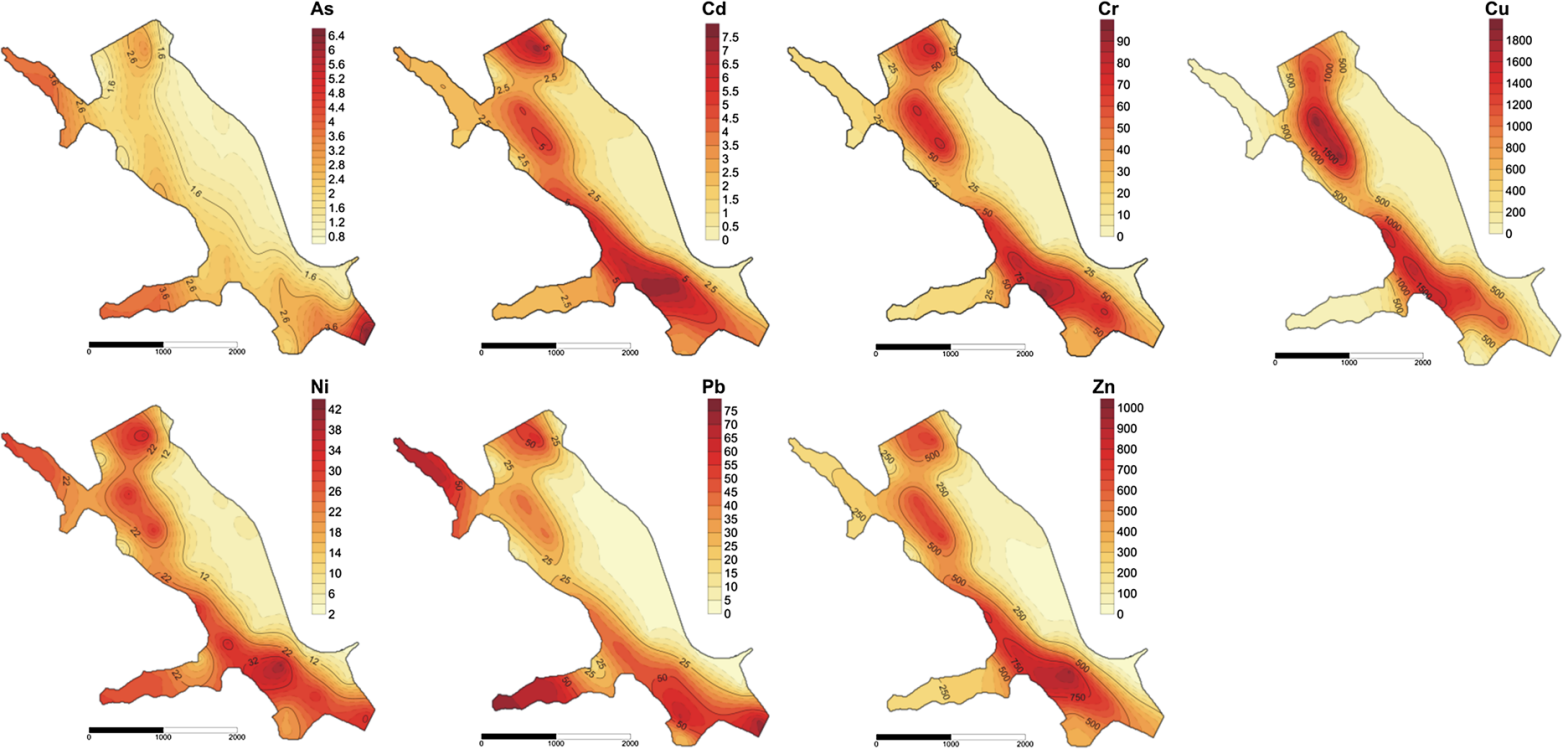
Spatial distribution of potential mobile fraction (PMF ∑1–3) of trace elements (mg/kg d.m.) in bottom sediments

It has been widely reported that trace elements in the exchangeable fraction (F1) are considered to be more mobile and bioavailable (MF, mobile fractions). Moreover, the potential mobile fraction (PMF) of elements, including non-residual fraction 1, 2 and 3 (PMF ∑1–3), is more bioavailable than the residual (fraction 4, RF), especially if the sediment condition was changed (Rinklebe and Shaheen 2014; Aguilar-Hinojosa et al. 2016). Elements associated with the residual fraction are stable, bound within a structure of some primary and secondary minerals, and they cannot have an impact on the water quality and organisms (Martínez-Santos et al. 2015; Aguilar-Hinojosa et al. 2016). The potential mobile fraction (PMF) ranged from 18 to 48% As, from 30 to 43% Cd, from 55 to 95% Cr, from 55 to 92% Cu, from 62 to 71% Ni, from 28 to 72% Pb and 47–62% Zn of the total content of the elements. However, the distribution of the mobile fraction MF (F1) was as follows: 0.4–0.6% As; 28–42% Cd; 0.2–2% Cr; 5–37% Cu; 11–20% Ni, 0.7–4% Pb and 26–34% Zn of the total content of the trace elements. In the study, different distributions of MF and PMF were found depending on the TOC content in the bottom sediments (Figs. 4, 5). Generally, a higher content of organic carbon in sediments contributes to the increase (Cu, Pb, Cr, Ni, Zn) and decrease (Cd, As) in the share of elements in the PMF ∑1–3. On the other hand, the higher TOC content in sediments reduced the binding of As, Cd, Cr, Cu, Pb and increased the binding of Ni, Zn in the mobile fractions (F1). Trace elements of an anthropogenic origin are present mainly in the first three fractions, while in the fourth fraction (RF), elements of a lithogenic origin are present (Baran and Tarnawski 2015). In the studies, it was found that the percentages of trace elements (Cu-81%, Cr-91%, Ni-71%, Zn, Cd, Pb-60%) bound in the PMF were higher than those of the RF (F4), which implies that these elements primarily come from anthropogenic inputs.

### Assessment of the actual and potential mobility of trace elements in the bottom sediments

Two guidelines were applied to assess the actual and potential mobility of elements from the sediments. The first was the risk assessment code (RAC) classification based on the percentage of trace elements in the MF (F1) (Singh et al. 2005; Kumar et al. 2014). The second guideline was the individual and global contamination factor (ICF and GCF). Both factors assess the potential mobility of trace elements from the sediments in relation to their retention time (Naji et al. 2010). A high ICF of elements shows a low retention time and a high potential risk to the environment (Namati et al. 2009). The ICF was calculated based on dividing the potential mobile fraction (PMF ∑1–3) by the residual fraction (F4):

$${\text{ICF}} = \frac{{{\text{PMF}}\sum {1 - 3} }}{{{\text{F}}4}}$$

In addition, the GCF for seven elements and for each sediment sample was calculated as:

$${\text{GCF}} = \sum {\text{ICF}}$$

According to the literature, MF (F1) is considered as the bioavailable fraction and it is used to assess the actual ecological risk (Table 2). Depending on the TOC content in the bottom sediments, medium and high risk for zinc was found; high, medium and low risk for copper; high and medium for cadmium; medium risk for nickel; low and no risk for chromium and lead; and no risk in the case of arsenic released from bottom sediments. Taking into account the RAC classification, the highest mobility of trace elements (Cd, Cr, Cu, Pb) from the bottom sediments to the aquatic environment was found in the samples with a low content of TOC (< 10 g/kg d.m.). In the sediments that contain more than 100 g/kg d.m. TOC, no and medium risk of trace element release from bottom sediments was observed (Table 2).

In Table 2, the ICF values of each trace element in the sediments are shown. The mean ICF of the sampling sites are ranged in the following order: Ni > Cd > Cr > Cu > Zn > As > Pb (low content of TOC); Cr > Cu > Ni > Pb > Cd > Zn > As (medium content of TOC); and Cr > Cu > Ni > Zn > Pb > Cd > As (high content of TOC). High values of ICE were found for chromium; however, the content of chromium in bottom sediments (∑ F1–F4 33.66 mg/kg) was relatively low, which indicates a low real risk of chromium for the aquatic environment. From the calculated ICF, Cu (medium and high TOC content), Cd and Ni (low TOC content) were potentially the most toxic elements for biota in the Rybnik reservoir. Arsenic posed the lowest risk to the water environment. The application of GCF is significant because it reflects the total potential risks posed by toxic elements to the water environment and to the biota (Ikeam et al. 2004). The values of GCF in the samples were 10.03 (low content of TOC); 33.07 (medium content of TOC) and 67.80 (high content of TOC). The highest values of GCF were found in the site sample characterised by the highest content of TOC: 6, 10, 17, 30 (Figs. 1, 2a, b). These points identify an extremely high potential risk to the aquatic environment due to trace element pollution in the case that the physicochemical parameters change.

### Ecotoxicity of bottom sediments

The Microtox test with *V. fischeri* is one of the most widely used biotests for bottom sediment toxicity due to high sensitivity and ecological relevance of bacteria (Zadrozhnaya et al. 2015; Rosado et al. 2016a; Tarnawski and Baran 2018). In the Microtox biotest, the luminescence of *V. fischeri* was between 24 and 95%. Slightly higher inhibition of light production in the bacteria after 5 min of exposure to contact with the sediment extracts was observed in the samples with a high (66 ± 15%) and middle (66 ± 21%) content of TOC then low (59 ± 13%) content of TOC. Regardless of the TOC content in the sediments, most of the samples (80%) were toxic to *V. fischeri.* The response of the test organisms was characterised by a low spatial variability, and the CV values were between 22 and 32%. In the studies, an analysis of the correlation between the content of trace elements in different fractions and toxicity to *V. fischeri* was carried out (Table 3). Mobile fractions of trace elements have a higher influence on toxicity (Rosado et al. 2016a). Gao et al. (2018) observed that not all of the content of trace elements in the sediments was affected by sediment toxicity, but the acid-soluble forms of those elements caused significant toxicity towards *V. fischeri.* In our studies, a positive correlation was found between all fraction of the elements (except F1 of As, F2 of Cu and F3 of Cd) and inhibition of luminescence in *V.* fischeri (Table 3). However, the correlations were not statistically significant, which may suggest that the mobile fraction (F1) and the potential mobile fraction (PMF) of trace elements are not an important source of sediment toxicity to the bacteria in the Rybnik reservoir. The studies of Jaiswal and Pandey (2018) found that heavy metal accumulated in the sediment inhibits enzyme activities; however, sediment rich in TOC had a relatively low toxicity, which was probably due to the reduced bioavailability of metals. Moreover, the toxicity is characterised by a direct hazard posed by the bottom sediment samples to living organisms connected with all chemical compounds and their mixtures (Rosado et al. 2016a). Therefore, our results imply that other chemical compounds and factors, which were not assessed in this study, can cause inhibition luminescence of bacteria.

**Table 3:** Correlation coefficients of trace element fractions and chemical, physical and ecotoxicological properties of sediments

Fraction	^a^TOC	^b^Cext	^c^Ckh	^d^Cfa	^e^Cnh	^f^DOC	Sand	^g^Mud	pH	^h^Eh	^i^LI
F1											
As	**0.37**	**0.56**	**0.46**	**0.56**	0.31	0.07	− 0.16	− 0.12	− 0.32	**0.39**	0.00
Cd	**0.79**	**0.76**	**0.79**	**0.75**	**0.78**	**0.87**	− 0.17	0.13	− 0.01	0.02	0.08
Cr	**0.64**	**0.56**	**0.68**	**0.55**	**0.65**	**0.78**	− 0.23	− 0.02	0.14	− 0.09	− 0.06
Cu	0.10	0.00	0.16	0.01	0.13	**0.36**	0.00	0.08	0.26	− 0.29	0.08
Ni	**0.82**	**0.77**	**0.77**	**0.77**	**0.81**	**0.91**	− 0.21	0.14	− 0.03	0.03	0.11
Pb	0.27	**0.54**	**0.45**	**0.54**	0.20	0.11	− 0.39	− 0.16	− 0.32	**0.38**	0.13
Zn	**0.80**	**0.70**	**0.76**	**0.69**	**0.81**	**0.90**	− 0.09	0.16	0.03	− 0.02	0.07
F2											
As	**0.43**	**0.48**	**0.43**	**0.48**	**0.41**	0.11	− 0.14	0.05	− 0.31	**0.35**	0.11
Cd	**0.85**	**0.78**	**0.76**	**0.77**	**0.84**	**0.92**	− 0.17	0.05	0.00	− 0.01	0.03
Cr	**0.54**	**0.40**	**0.50**	**0.39**	**0.56**	**0.63**	0.08	0.16	0.13	− 0.11	0.02
Cu	**0.43**	0.33	**0.46**	0.32	**0.45**	**0.65**	0.02	0.15	0.19	− 0.23	0.03
Ni	**0.83**	**0.79**	**0.78**	**0.78**	**0.82**	**0.91**	− 0.17	0.08	0.02	− 0.02	0.01
Pb	**0.44**	**0.65**	**0.55**	**0.65**	**0.38**	0.21	− 0.33	0.01	− 0.24	0.34	0.03
Zn	**0.83**	**0.73**	**0.76**	**0.73**	**0.84**	**0.92**	− 0.10	0.11	0.11	− 0.13	0.00
F3											
As	**0.73**	**0.89**	**0.71**	**0.90**	**0.67**	**0.56**	− 0.35	0.07	− 0.30	0.33	0.09
Cd	**0.79**	**0.67**	**0.75**	**0.66**	**0.80**	**0.78**	0.07	0.09	− 0.08	0.11	0.01
Cr	**0.84**	**0.74**	**0.76**	**0.73**	**0.85**	**0.94**	− 0.14	0.10	0.08	− 0.09	− 0.01
Cu	**0.71**	**0.60**	**0.69**	**0.59**	**0.72**	**0.91**	− 0.06	0.10	0.23	− 0.26	− 0.05
Ni	**0.90**	**0.93**	**0.87**	**0.93**	**0.88**	**0.83**	− 0.16	0.12	− 0.12	0.13	0.06
Pb	**0.84**	**0.92**	**0.81**	**0.92**	**0.80**	**0.74**	− 0.32	0.13	− 0.09	0.16	− 0.05
Zn	**0.86**	**0.79**	**0.83**	**0.78**	**0.85**	**0.86**	− 0.02	0.10	0.00	0.01	− 0.01
F4											
As	**0.79**	**0.82**	**0.88**	**0.81**	**0.77**	**0.90**	− 0.17	− 0.01	− 0.11	0.10	0.17
Cd	**0.83**	**0.78**	**0.79**	**0.77**	**0.83**	**0.88**	− 0.11	0.06	− 0.13	0.14	0.06
Cr	0.10	0.14	0.01	0.15	0.09	0.18	0.08	− 0.08	− 0.32	0.32	0.43
Cu	0.16	0.11	0.24	0.10	0.17	0.28	0.19	0.02	− 0.10	0.09	0.13
Ni	**0.65**	**0.54**	**0.60**	**0.53**	**0.66**	**0.73**	− 0.13	− 0.07	− 0.17	0.18	0.08
Pb	**0.85**	**0.80**	**0.88**	**0.79**	**0.84**	**0.90**	− 0.11	− 0.03	− 0.06	0.07	0.10
Zn	**0.78**	**0.68**	**0.74**	**0.67**	**0.79**	**0.83**	− 0.01	0.14	− 0.07	0.08	0.06

^a^Total organic carbon, ^b^carbon in the extract, ^c^carbon of humic acids, ^d^carbon of fulvic acids, ^e^non-hydrolysing carbon, ^f^dissolved organic carbon, ^g^mud (silt + clay), ^h^redox potential, ^i^luminescence inhibition of *V. fischeri*

Bold: significant at *p* ≤ 0.05

0 < *r* < 0.3 very low correlation; 0.3 ≤ *r* < 0.5 low correlation; 0.5 ≤ *r* < 0.7 medium correlation; 0.7 ≤ *r* < 0.9 strong correlation; 0.9 ≤ *r* < 1 very strong correlation

### Correlation coefficient and PCA analysis

**Fig. 6:**
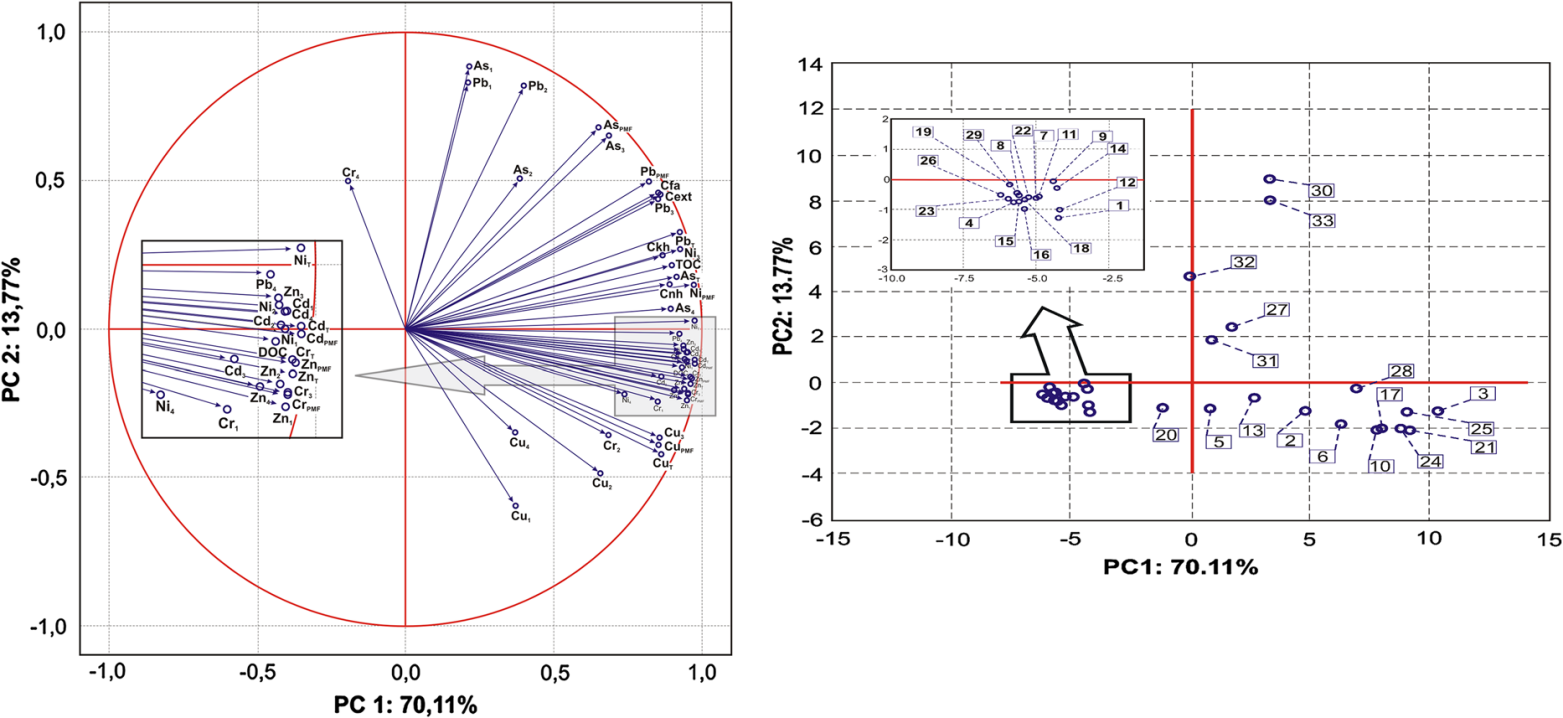
PCA applied to the results of trace element content (F1, F2, F3, F4, PMF and T (∑fraction 1–4) and fraction of organic matter in the bottom sediments

Potential factors influencing the chemical fractions of trace elements are shown in Table 3 as well as Fig. 6a, b. In these studies, the pH, redox potential and particle size showed no significant correlation with the content of trace elements regardless of the trace element fractions. A significant positive correlation was found only for the Eh and content fraction F1 of As, Pb, fraction F2 of As. The absence of a more significant correlation between the above parameters suggests that the pH, redox potential and partial size do not play a dominant role in the mobility and sorption of trace elements. Generally, a significant positive correlation was found between the content of organic matter fraction and the distribution of trace elements between the analysed fractions (Table 3). A positive correlation but no significant was observed between the content of As and Pb (F1, F2), Cr (F4), Cu (F1, F2, F4) and the content of the different organic matter fraction. The results would suggest that the transformation and sources of organic matter play an important role in the behaviour of trace elements in the bottom sediments of the Rybnik reservoir. The relationships between TOC and trace element contents have often been associated with the adsorption and complexation of elements by organic matter of both autochthonous and terrestrial origin (Yang et al. 2011).

The conducted PC analysis allowed to observe several interesting relationships between the investigated variables (Fig. 6). The PCA extracted two PC explaining 83.88% of the total variance of the dataset. PC1 explaining 70.11% of the total variance and has strong positive loadings (> 0.70) on TOC, Ckh, Cnh, DOC, ∑F1–4, F4 (except Cu, Cr), PMF (except As, Pb), F3 (except As, Pb), F2 (except As, Pb) and F1 (except As, Cu and Pb) of trace elements. PCA analysis also found strongly positive correlations between Cext, Ckh, Cfa, Cnh, DOC (*r* = 0.78–0.98 at *p* ≤ 0.05) and TOC. The results indicated that PC1 covering the fraction of organic matter and trace element content in the bottom sediment have the same origin of both natural and anthropogenic sources. Moreover, the high loading for the fraction of organic matter indicates the importance of the organic matter to the binding of trace element ions in the analysed sediments. On the other hand, a strong significant correlation between TOC and the content of trace elements in different fractions indicated that the mineralisation of organic matter and the associated release of trace elements are an important secondary source of elements in the Rybnik reservoir (Table 3, Fig. 6). The second component (PC2) accounted for 13.77% of the total variance with a strong positive loading (> 0.70) for Cext, Cfa and As, Pb (F1, F2, F3, PMF) (Fig. 6). The combination of trace elements (As, Cu, Pb) in the PC2 suggested their other origins and sources, independent of organic matter transformations. The spatial distribution of PMF and F1 of Cu, As, Pb in the bottom sediments (Figs. 4, 5) confirms the above relationship.

Moreover, the correlation analysis and PCA found very strong and strong correlations between DOC and Zn, Cd, Ni (F1, F2, F3, F4, ∑F1–4, PMF), Cr (F1, F2, F3, ∑F1–4, PMF) As (F4, ∑F1–4), Pb (F3, F4, ∑F1–4, PMF) and Cu (F3, ∑F1–4, PMF). Medium and low correlations were observed between DOC and Cu (F1, F2, F4) and As (F3, PMF). This implies that the mobility of these trace elements is bound to DOC—independent of organic matter, which could play an important role in the complexation of these elements. Stable forms of organic matter (TOC, Cnh, Cha) had stronger and medium correlations with F3, F4, F2 of As and Pb; F1, F2, F3, PMF of Ni; F3, PMF of Cu and low with F2 of Cu; F1 of As, Pb. Finally, the Cext and Cfa had a stronger and medium positive correlation with F1, F3, PMF of Pb and As (Fig. 6). The PCA analysis also confirmed the previously observed significant difference between the behaviour of trace elements in the sediment samples with a high, medium and low content of TOC. In Fig. 6b, it can clearly be found that the sediment samples with the highest content of TOC (28, 17, 24, 10, 21 3, 25) and a lower share of trace elements in MF are distributed towards the positive side of PC1. On the other hand, a negative relationship with PC1 was demonstrated for sediment samples with a low TOC content and a high share of trace elements in F1 (12, 11, 22, 1, 19, 18, 8, 7, 16, 29, 4, 23, 26, 15) (Fig. 6). This means that the high content of TOC significantly decreased the mobility and bioavailability of trace elements in the bottom sediments of the Rybnik reservoir. Moreover, it can be said that in the above sediment samples, similar factors or sources are responsible for the content and transformation of organic matter and the distribution of trace elements in the different fractions. However, the points representing medium content of TOC, and especially points 30, 31, 32, 33, differed considerably from the above groups. These points are located in very close proximity to the western part of the reservoir and beyond its main part (31, 32, 33) (Fig. 1). The results suggested that other factors and sources influence the mobility of trace elements and the content of different organic carbon fractions in these sediment samples. The main sources of trace elements in the bottom sediments of the Rybnik reservoir are the metallurgical industry, combustion of coal and dry precipitation (Baran and Tarnawski 2015). It should be emphasised that in the region—the Silesian Voivodeship where the Rybnik reservoir is located—the emission of dust and gas pollution accounts for 19% of the total emissions in Poland. Atmospheric Pb and As deposition may be an important source of both elements in the bottom sediments. Moreover, organic matter and trace elements enter the Rybnik reservoir together with municipal wastewater, industrial sewage discharged by the Rybnik power plant and long-range transport associated with the contaminated water of the Ruda river (Loska and Wiechuła 2003). Under oxidising conditions, the mobility of trace elements from the bottom sediments increases. Loska and Wiechuła (2003) found that the inlet part of the reservoir is characterised by increased oxidising conditions in the bottom sediments compared to other parts of the reservoir. Temperature is also an important parameter controlling the behaviour of trace elements and the transformation of organic matter in the aquatic environment. Fonseca et al. (2013) observed an influence of higher temperature on the intensified growth of the bacterial population and thus a quicker degradation of organic matter. In the Rybnik reservoir, the inflow of heated water from the power plant to the reservoir can potentially affect the transformation of organic matter and increase the solubility of trace elements from the bottom sediments (Kostecki et al. 2017).

It is widely known that a strong correlation between the elements indicates that they have a common source and identical behaviour during transport. We observed that the concentration of trace elements (pairs of Zn, Cd, Ni, Cr and pairs of As, Pb) was strongly, positively correlated (Fig. 6). However, a slightly different behaviour was observed for the copper content in the bottom sediments (Figs. 4, 5, 6). In our previous studies, it has been shown that eluting Cu from the cooling system of the Rybnik power plant is an important source of copper in the bottom sediments (Baran and Tarnawski 2015). Other authors also found that effluents from power plants that use copper alloys in the heat exchangers of their cooling systems discharge copper into receiving waters (Bojakowska and Krasuska 2014).

Apart from the results of the PCA analysis (Fig. 6), a similar spatial distribution of TOC, PMF and MF of trace elements in the bottom sediments was also observed in the studies (Figs. 2, 4, 5). The spatial layout of the above parameters constitutes an outcome of three factors: water movement, trophic level and anthropogenic impact (Kostecki 2004; Kostecki et al. 2017). Changes in the water temperature cause the vertical circulation of water masses, which leads to varying conditions at the place where the reservoir water and the water that feeds the Ruda river mix. The phenomena of the mixing and movement of water masses in the reservoir resulting from the dumping of industrial waters (tributary of heated water from the power plant) are stronger than natural factors (tributary of the Ruda river) (Kostecki et al. 2017). The resulting water circulation in the reservoir contributes to the creation of turbulence and reverse currents in the horizontal layout of the reservoir. As a result of the specific movement of water inside the reservoir, a zone was created, which exhibits the highest content of TOC and the potentially mobile as well as mobile fraction of trace elements. This zone has been located in the western as well as the north-western section of the reservoir (Figs. 2, 4, 5). The potential sources of trace elements, as well as allochthonous organic carbon, may result from recreational activities (hotel, beaches, marinas, stud farm) as well as transport (no. 920 Voivodeship road) located in close vicinity to the western part of the reservoir.

## Discussion

Knowledge of the fraction of trace elements in the bottom sediments is a key to understanding both their geochemical mobility and their ecotoxicological impact. On the other hand, different fractions of organic matter are known to regulate the behaviour of trace elements in the water ecosystem (Calace et al. 2006; Yang et al. 2011). The interactions of trace elements with a variety of organic substances (fulvic acid, humic acid, non-hydrolysing carbon, DOC) play an important role in the distribution, bioavailability and protection of aquatic organisms against the toxicity of an excessive content of trace elements (De Schamphelaere et al. 2005; Yang et al. 2011; Fonseca et al. 2013; Smith et al. 2014; Bai et al. 2018). Trace elements bound on humic substance are relatively immobile, and the functional groups: carboxylic (–COOH), phenolic (–OH) and amino acid residues (amine, amide, sulphur groups) are mainly involved in the formation of element-humic complexes; however, this sorption behaviour is strongly impacted by the pH and ionic strength (Smith et al. 2014; Boguta and Sokołowska 2016; Bai et al. 2018). Therefore, the identification of these interactions is useful for the prediction of the fate and transport of trace elements in the sediments as well as a justified pollution risk assessment (Boruvka and Drábek 2004; Calace et al. 2006; Boguta and Sokołowska 2016). We found a significant correlation between the different fractions of trace elements and all organic matter fractions in the bottom sediments. However, our results indicated that these relationships are complex. The highest value of correlation coefficients was observed for different fractions of trace elements and the content of DOC in the bottom sediments. Several authors found that DOC plays an important role in mitigating the mobility of trace elements to water organisms (De Schamphelaere et al. 2005; Smith et al. 2014). DOC can decrease the trace element toxicity by a complex with element cations as well as making them less bioavailable. De Schamphelaere et al. (2005) and Al-Reasi et al. (2012) have reported that DOC reduces Cu toxicity to the *Daphnia magna*. In this study, high toxicity of bottom sediments to *V. fischeri* was found. However, despite the high content of the mobile fraction and the potential mobile fraction of trace elements, the correlation between the content of trace elements and the response of bacteria was insignificant (Table 3). These results may be an effect of the complexation of trace elements with DOC, which makes them less bioavailable for organisms. These complexes are too large and too polar to be able to diffuse through cell membranes of organisms. Moreover, it was indicated that DOC of a terrestrial origin (plant-derived) is more aromatic and more effective in decreasing the toxicity of metals than DOC of an autochthonous origin (Al-Reasi et al. 2012; Smith et al. 2014). In our previous studies, a high C/N ratio in sediments from the Rybnik reservoirs was found, which suggests that the deposition of terrigenous material is dominant in the reservoir (Baran et al., 2017). On the other hand, a significant share of DOC in the binding of trace elements may be related to the intensive eutrophication process occurring in the Rybnik reservoir (Kostecki et al. 2017). A eutrophic environment enhances the production of autochthonous dissolved organic matter (DOC). Dong et al. (2018) found that in this condition, the complexing capacity of DOC for Cd increases due to the elevated DOC content during algal bloom; however, the increased complexing capacity reduces the potential bioavailability of Cd. Among the humic substances, the highest values of correlation coefficients with a different fraction of trace elements were shown for Cnh (non-hydrolysing carbon) (Table 3). The high affinity between trace elements and Cnh occurred because this fraction is more stable and stronger in the case of complex inorganic pollution (Durand et al. 2005; Xu et al. 2017). High sorption capacities for organic pollutants are also characterised by non-hydrolysing carbon instead of humic acids and fulvic acids (Huang et al. 2003; Yang et al. 2011). Cnh is the insoluble component of organic matter, and vin comparison to other humic substances, it is relatively resistant to decomposition (Bai et al. 2018; Xu et al. 2017; Hayes et al. 2017). Aliphatic hydrocarbons, which occur in lipids, waxes, cuticular materials, cutin/cutan and suberin/suberin, are major components of non-hydrolysing carbon (Hayes et al. 2017; Xu et al. 2017). There are many studies on the complexation of trace elements with fulvic and humic acids in soils and bottom sediments (Schnitzer and Kerndorf 1981; Donisa et al. 2003; Boruvka and Drábek 2004; Yang et al. 2011; Smith et al. 2014; Boguta and Sokołowska 2016). Both humic and fulvic acids consist of aliphatic and aromatic carbon structures; however, humic acid contains carbon with a larger molecular size. The carbon structure of fulvic acid has a lower molecular weight and more oxygen—containing less functional groups than humic acid (Derrien et al. 2017). Donisa et al. (2003), as well as Boruvka and Drábek (2004), have shown that fulvic acid is generally the main humic fraction reacting with trace elements in soil and bottom sediments. Those studies found a higher content of Cfa and generally higher values of the correlation coefficients between different fractions of trace elements. Schnitzer and Kerndorf (1981) suggested that in the pH range of 5–7, trace elements have a higher tendency to form a water-insoluble complex with fulvic acid, which could lead to the accumulation of such complexes in sediments or soil.

## Conclusion

We conclude that the Rybnik reservoir is enclosed and has a low rate of water exchange. These conditions are suitable for the accumulation of organic matter in the bottom sediments. We found that the most refractory fraction of organic matter—Cnh—dominated in the sediments. The content of organic matter fractions are arranged in the following order: Cnh > Cfa > Cha > DOC. It has to be noted that both the content of Cha and Cfa in the content of TOC decreased along with an increase in the TOC content. However, the share of Cnh in the content of TOC increased along with an increase in the TOC content. An intensive accumulation of poorly decomposing macrophyte tissues in the bottom sediments, which decreased the contribution of humus substances in the TOC content, is responsible for the above relation. Moreover, municipal and industrial wastewater as well as the Ruda river, which are one of the main water sources of the Rybnik reservoir, have a high level of TOC and trace elements. The water temperature significantly affects the number of processes occurring in the reservoir, including the increase in the mobility of trace elements. The sources of trace elements in the bottom sediments can also be connected with an intensive anthropogenic input. Moreover, in the bottom sediments from the Rybnik reservoir, the results indicated that the organic matter fractions and their relationship with trace elements are more important than the physical–chemical parameters. The higher content of TOC in the sediments increased the share of elements in the potential mobile fraction and, at the same time, decreased the binding of elements in the mobile fractions. We found that Cu, Cd and Ni (low TOC content) are potentially the most toxic elements for biota in the Rybnik reservoir. A stronger correlation was observed between most of the fraction of trace elements and Cnh, while for Cfa and Cha, the above correlations were slightly weaker. However, the highest value of correlation coefficients was found for different fractions of trace elements and DOC content in the bottom sediments. These results suggested that the complexation of trace elements with DOC makes them less bioavailable for organisms. To sum up, the identification of the fraction distribution of trace elements and their relations with organic matter in the bottom sediments were the important steps in determining their mobility, potential bioavailability and toxicity to the aquatic environment.
